# Clues on Syntenic Relationship among Some Species of Oryzomyini and Akodontini Tribes (Rodentia: Sigmodontinae)

**DOI:** 10.1371/journal.pone.0143482

**Published:** 2015-12-07

**Authors:** Pablo Suárez, Cleusa Yoshiko Nagamachi, Cecilia Lanzone, Matias Maximiliano Malleret, Patricia Caroline Mary O’Brien, Malcolm Andrew Ferguson-Smith, Julio Cesar Pieczarka

**Affiliations:** 1 Laboratório de Citogenética, Centro de Estudos Avançados da Biodiversidade, ICB, UFPa, Belém, Pará, Brazil; 2 Laboratorio de Genética Evolutiva, IBS, FCEQyN, CONICET-UNaM, Posadas, Misiones, Argentina; 3 Cambridge Resource Centre for Comparative Genomics, Department of Veterinary Medicine, University of Cambridge, Cambridge, United Kingdom; 4 CNPq Researchers, Brasilia, Brazil; Universita degli Studi di Roma La Sapienza, ITALY

## Abstract

Sigmodontinae rodents represent one of the most diverse and complex components of the mammalian fauna of South America. Among them most species belongs to Oryzomyini and Akodontini tribes. The highly specific diversification observed in both tribes is characterized by diploid complements, which vary from 2n = 10 to 86. Given this diversity, a consistent hypothesis about the origin and evolution of chromosomes depends on the correct establishment of synteny analyzed in a suitable phylogenetic framework. The chromosome painting technique has been particularly useful for identifying chromosomal synteny. In order to extend our knowledge of the homeological relationships between Akodontini and Oryzomyini species, we analyzed the species *Akodon montensis* (2n = 24) and *Thaptomys nigrita* (2n = 52) both from the tribe Akodontini, with chromosome probes of *Hylaeamys megacephalus* (2n = 54) of the tribe Oryzomyini. The results indicate that at least 12 of the 26 autosomes of *H*. *megacephalus* show conserved synteny in *A*. *montensis* and 14 in *T*. *nigrita*. The karyotype of *Akodon montensis*, as well as some species of the *Akodon cursor* species group, results from many chromosomal fusions and therefore the syntenic associations observed probably represent synapomorphies. Our finding of a set of such associations revealed by *H*. *megacephalus* chromosome probes (6/21; 3/25; 11/16/17; and, 14/19) provides phylogenetic information for both tribes. An extension of these observations to other members of Akodontini and Oryzomyini tribes should improve our knowledge about chromosome evolution in both these groups.

## Introduction

Sigmodontinae rodents represent one of the most diverse and complex components of the mammalian fauna of South America, currently compost by around 380 species belonging to 74 genera. With the exception of 11 *incertae sedis* genera, all species of the subfamily are grouped into nine tribes (Abrotrichini, Akodontini, Ichthyomyini, Oryzomyini, Phyllotini, Reithrodontini, Sigmodontini, Thomasoyini, and Wiedomyini) [1, 2]. About 60% of the Sigmodontinae belong to tribes Oryzomyini and Akodontini, which contain 120 and 106 recognized species, respectively [1].

The highly specific diversification observed in both tribes is characterized by a remarkable variability of diploid numbers, which vary from 2n = 16 to 86 in Oryzomyini and 2n = 10 to 54 in Akodontini [3, 4]. Given this diversity, a hypothesis about the origin and evolution of their chromosomes depends on the correct establishment of synteny analyzed in a suitable phylogenetic framework. Comparative analysis of G-banded chromosomes enabled the identification of numerous chromosomal alterations in Akodontini [5, 6], which has led to the elaboration of phylogenetic hypotheses that explain this chromosomal variability by the occurrence of centric fusions, tandem fusions and inversions. However, there are many chromosomal differences that cannot be explained solely by G-banding techniques and molecular cytogenetic techniques are required.

Chromosome painting has been particularly useful in solving problems of chromosome evolution in old world rodents (e.g. [7–10]). However, in new world rodents there have been only a few studies in Sigmodontinae. Among them, Swier et al. [11] used *Sigmodon* (Sigmodontini tribe) probes for cross-species FISH on nine species of the genus *Sigmodon*, which allowed them to postulate an ancestral *Sigmodon* karyotype and three diploid number reductions. Likewise, cross-species chromosome painting with *Oligoryzomys moojeni* chromosome probes allowed Di-Nizo et al. [12] to uncover the complex and extensive chromosome re-patterning involved in the diversification of seven *Oligoryzomys* species. Additionally, conventional and reverse chromosome painting experiments in four species of *Akodon* with different diploid numbers allowed Ventura et al. [13] to characterize a sequence of tandem fusion events involved in the chromosomal evolution of species in the *Akodon cursor* group with low diploid numbers, previously suggested by their G-banding patterns [14, 15].

Studies involving inter-generic comparison have uncovered several clues of conserved synteny among five Akodontini species (*Akodon cursor*, *A*. *montensis*, *A*. *paranaensis*, *A*. *serrensis*, *Necromys lasiurus*, and *Thaptomys nigrita*) and one Oryzomyini species (*Oligoryzomys flavescens*) using *Mus musculus* chromosome-specific probes [16, 17]. Finally, Nagamachi et al. [18] developed species-specific chromosome probes for *Hylaeamys megacephalus* that disclosed major karyotypic reconstruction in *Cerradomys langguthi* (Oryzomyini species). These studies demonstrate the value of chromosome painting in understanding chromosome evolution in new world rodents.

In order to extend our knowledge of the phylogenetic relationships between Akodontini and Oryzomyini species, we analyzed *A*. *montensis* (2n = 24) and *T*. *nigrita* (2n = 52) of the tribe Akodontini, with probes from *H*. *megacephalus* (2n = 54) of the tribe Oryzomyini. This approach allowed us to identify shared and unique syntenic associations in each of these species.

## Material and Methods

### Ethics Statement

Animals collected during this study were handled following procedures recommended by the American Society of Mammalogists [19], and were made under specific collecting licenses from the Ministerio de Ecologia y Recursos Naturales Renovables de la Provincia de Misiones, Argentina (9910-00136/14; headed by CL as principal investigator) and Instituto Chico Mendes de Conservação da Biodiversidade, Brazil (N°13248; headed by JCP as principal investigator).

JCP has a permanent field permit, number 13248 from “Instituto Chico Mendes de Conservação da Biodiversidade”. The Cytogenetics Laboratory from UFPa has permit number 19/2003 from the Ministry of Environment for sample transport and permit 52/2003 for using the samples for research. The Ethics Committee (Comitê de Ética Animal da Universidade Federal do Pará) approved this research. The rodents were maintained in the lab with food and water, free from stress, until their euthanasia by IP injection of buffered and diluted barbiturates after local anesthetic.

### Sample and chromosomes

*Akodon montensis* male and female adults and *Thaptomys nigrita* female adults were collected in several localities of Misiones Province, Argentina (Iguazú S25°67’67”/W54°44’66”; San Ignacio S27°16’82”/O55°34’45”; Puerto Esperanza S25°59’20”/W54°38’35”; Uruguaí S25°51’22”/W54°10’; and, Candelaria S27°10’6”/W55°20’40”) using “Sherman” and “Tomahawk” live-traps. The skulls and skins were preserved and deposited in the Mastozoological collection housed in the Laboratorio de Genética Evolutiva, Instituto de Biología Subtropical, Posadas, Misiones, Argentina. Chromosome preparations were obtained from bone marrow following Ford and Hamerton [20], with some modifications. Gross chromosomal characterization was established by C-Banding [21], and G-Banding [22].

### Probes and FISH

Each whole chromosome probe is the product of PCR amplification of copies of the same chromosome. The templates were chromosomes of *H*. *megacephalus* sorted at the University of Cambridge using a Dako flow cytometer [18]. The DNA sequence of the probes used for FISH is unknown and they were not molecularly characterized. Primary PCR products were labeled either with biotin-16-dUTP (Boehringer Mannheim), FITC-12-dUTP, or Cy3-dUTP (Amersham) by a second round of DOP-PCR with 6MW primers [23, 24]. The DNA fragment size of probes were checked on 1% agarose gel electrophoresis (100V, 40 min), and had an average size of 0,2–0,8 kb. As some *H*. *megacephalus* chromosome pairs could not be separated from others by cytometry (pairs 9/10, 13/22, and 16/17), the combined probes recognize homologous regions of more than one chromosome pair. Fluorescent *In situ* Hybridization (FISH) in metaphase chromosomes of *A*. *montensis* and *T*. *nigrita* with chromosome-specific probes was performed as previously described [25]. Briefly, slides were pretreated with pepsin solution (50 ug/mL in 0,01M HCl) at room temperature for 5 min, dehydrated progressively with an ethanol series (70%, 90%, and 100%) 4 min each, and incubated for at least two hours at 65°C. DNA of mitotic chromosomes was denatured at 62°C with 70% formamide/0,6xSSC for 60 seconds, and quickly stopped with 4°C 70% ethanol for 4 min, followed by another ethanol series of dehydration. About 300 ng of labeled DNA probe was dissolved in 15 uL of hybridization buffer (50% deionized formamide/2xSSC/12,5% dextran sulfate/50mM phosphate buffer solution), and heated at 75°C for 15 min before being applied to the slides. Hybridization reactions were performed at 77% stringency for 48-72h at 37°C in a humid chamber. Post-hybridizations washes were performed in 50% deionized formamide/1xSSC and 2xSSC for 10 min each at 42°C. Indirect detection of biotinylated probes was made with avidin-Cy3 or avidin-FITC antibodies. DAPI stained chromosomes and fluorescent hybridization signals were captured and digitalized using a cooled CCD camera (Axiocam MRm), coupled to a Zeiss Axiophot microscope with Axiovision software.

## Results

**Fig 1:**
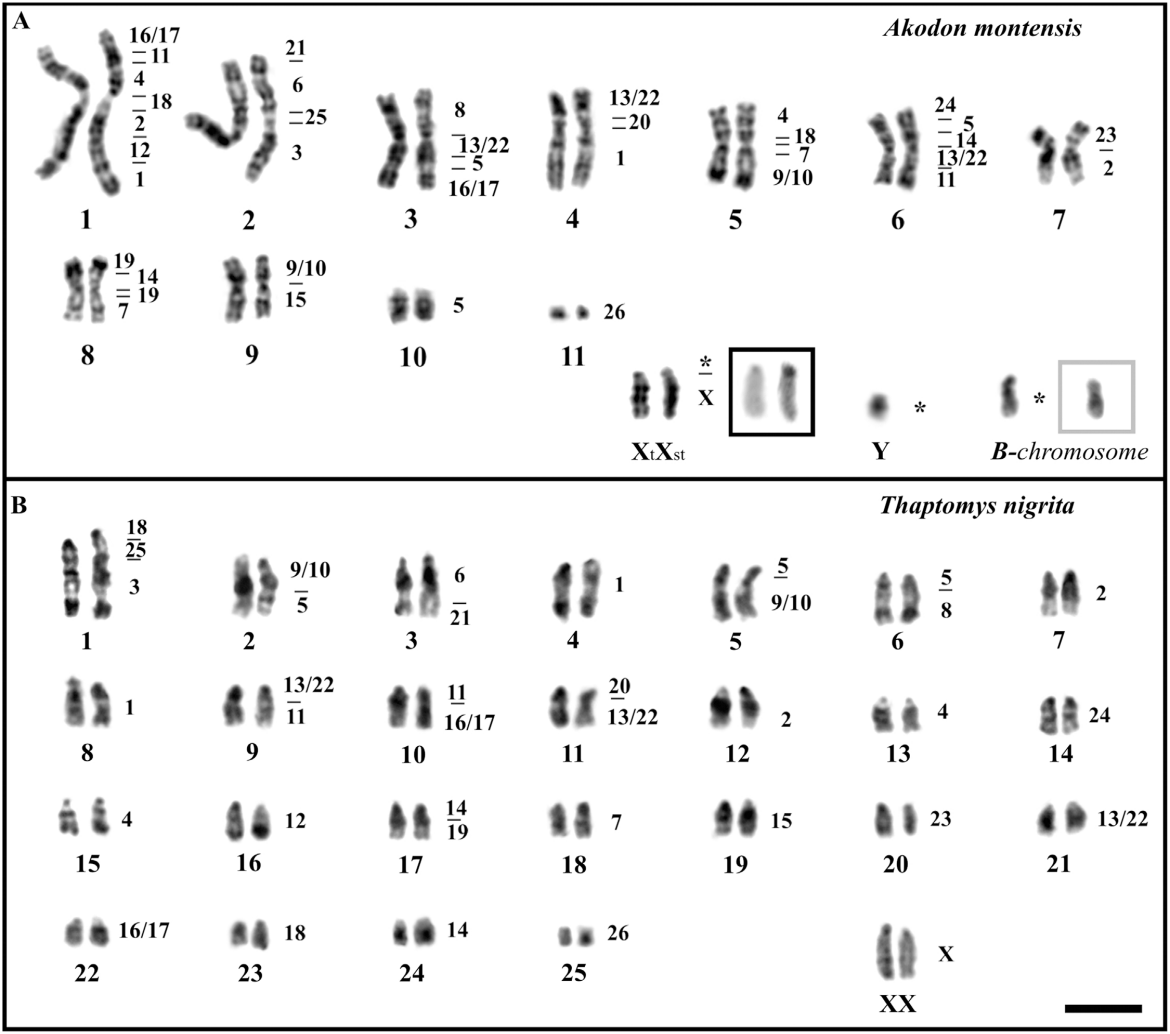
Chromosome complements of *Akodon montensis* and *Thaptomys nigrita* with regions of homology to chromosome-specific probes of *Hylaeamys megacephalus*. A-B: G-banded karyotypes. Black-square: C-banded X-chromosomes. Grey-square: C-banded B-chromosome. Bar = 10 μm.

*Akodon montensis* has 2n = 24 (FN = 42), mostly metacentric chromosomes, except the submetacentric pair 2 and the acrocentric pair 10. X and Y sex chromosomes also showed acrocentric morphology, with the exception of a polymorphic subtelocentric X chromosome (Fig 1A). The B-chromosome observed in this species is submetacentric (Fig 1A). *Thaptomys nigrita* has 2n = 52 (FN = 52), the metacentric pair 25 being the only biarmed chromosome pair (Fig 1B). The C-banding technique shows that *A*. *montensis* chromosomes are characterized mainly by centromeric and telomeric constitutive heterochromatin (CH). The acrocentric X chromosome has centromeric CH and the subtelocentric X has an additional heterochromatic block in the short arm (Fig 1A). The B-chromosome in turn shows CH on the centromere and has an interstitial block on the long arm (Fig 1A). In *T*. *nigrita* the CH is restricted to centromeric regions.

**Fig 2:**
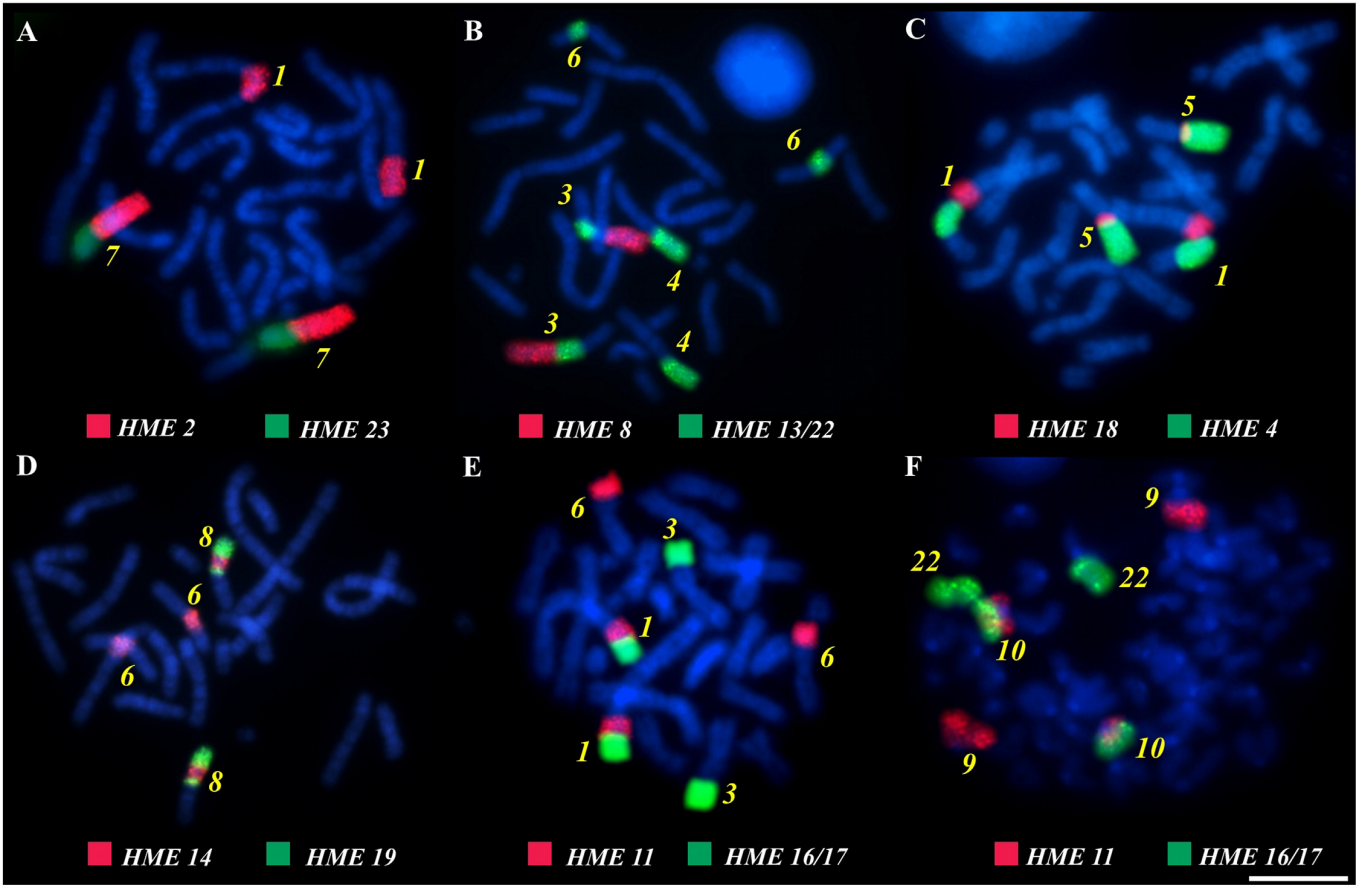
Hybridization *in situ* experiments with chromosome-specific probes of *Hylaemys megacephalus* hybridized to metaphase chromosomes of *Akodon montensis* (a, b, c, d and e) and *Thaptomys nigrita* (f) showing syntenic associations. Yellow numbers indicating each matching chromosome pair on target species. Bar = 10 μm.

All 24 whole chromosome probes of *Hylaeamys megacephalus* (1–8, 9/10, 11–12, 13/22, 14–15, 16/17, 18–21, 23–26, and X) were used to map chromosome homologies (Fig 1; S1 and S2 Figs) and positive and reproducible hybridization signals were observed in at least one chromosome region of both *A*. *montensis* and *T nigrita* (Fig 2). Table 1 summarizes these results. No hybridization signals were obtained for the Y chromosome (for which we have no *H*. *megacephalus* Y chromosome probe), for the short arm of the subtelocentric X chromosome of *A*. *montensis*, or for the B chromosome in the *A*. *montensis* female specimen (Fig 1).

**Table 1:** Total number and location of FISH signals observed on *Akodon montensis* and *Thaptomys nigrita* with specific-chromosome probes of *Hylaeamys megacephalus*.

*Hylaeamys megacephalus* Chromosome probes	*Akodon montensis*		*Thaptomys nigrita*	
	N° signals	Chromosome location	N° signals	Chromosome location
**1**	2	1q distal; 4q	2	4; 8
**2**	2	1q interstitial; 7q	2	7; 12
**3**	1	2q	1	1 interstitial and distal
**4**	2	1p proximal; 5p distal	2	13; 15
**5**	3	3q interstitial;6p interstitial; 10	3	2 distal; 5 proximal; 6 proximal
**6**	1	2p	1	3 proximal and interstitial
**7**	2	5q proximal; 8q	1	18
**8**	1	3p	1	6 distal
**9/10**	2	5q; 9p	2	2 proximal; 5 distal
**11**	2	1p interstitial; 6q distal	2	9 distal; 10 proximal
**12**	1	1q interstitial	1	16
**13/22**	3	3q proximal; 4p distal; 6q proximal	3	9 proximal; 11 interstitial and distal; 21
**14**	2	6p proximal; 8p interstitial	2	17 proximal; 24
**15**	1	9q	1	19
**16/17**	2	1p distal; 3q distal	2	10 distal; 22
**18**	2	1q proximal; 5p proximal	2	1 proximal; 23
**19**	2	8p distal; 8q proximal	1	17 distal
**20**	1	4p proximal	1	11 proximal
**21**	1	2p distal	1	3 distal
**23**	1	7p	1	20
**24**	1	6p distal	1	14
**25**	1	2q proximal	1	1 proximal
**26**	1	11	1	25
**X**	1	X (Xq*)	1	X

* subtelocentric X chromosome.

Twelve of the 24 whole chromosome probes show conserved synteny in *A*. *montensis* (*H*. *megacephalus* chromosomes 3, 6, 8, 12, 15, 20, 21, 23–26, and X) and 14 in *T*. *nigrita* (*H*. *megacephalus* chromosomes 3, 6–8, 12, 15, 19–21, 23–26 and X). Of these, seven chromosomes of *H*. *megacephalus* are preserved as unique linkage groups in *T*. *nigrita* (five with the same morphology: 7, 12, 15, 26 and the X chromosome; and two with a different morphology in *H*. *megacephalus*: pairs 23 and 24). Only two chromosomes of *H*. *megacephalus* (pairs 26 and X) were preserved as a discrete unit in *A*. *montensis* (pairs 11 and X). Thus, only the smallest pair in each of the three karyotypes and the X chromosome are wholly conserved between the three species.

More than one signal was observed using the remaining whole chromosome probes. Chromosome probes of *H*. *megacephalus* pairs 5 and 13/22 gave the highest number of signals, complementary to three regions of three chromosome pairs in *A*. *montensis* (pairs 3, 6 and 10, and pairs 3, 4 and 6, respectively) and *T*. *nigrita* (pairs 2, 5 and 6, and pairs 9, 11 and 21, respectively). Finally, the remaining *H*. *megacephalus* chromosome probes shared homology with 2 chromosome regions in *A*. *montensis* (probes of pairs 1, 2, 4, 7, 9/10, 11, 14, 16/17, 18, and 19) and in *T*. *nigrita* (probes of pairs 1, 2, 4, 9/10, 11, 14, 16/17, and 18).

**Fig 3:**
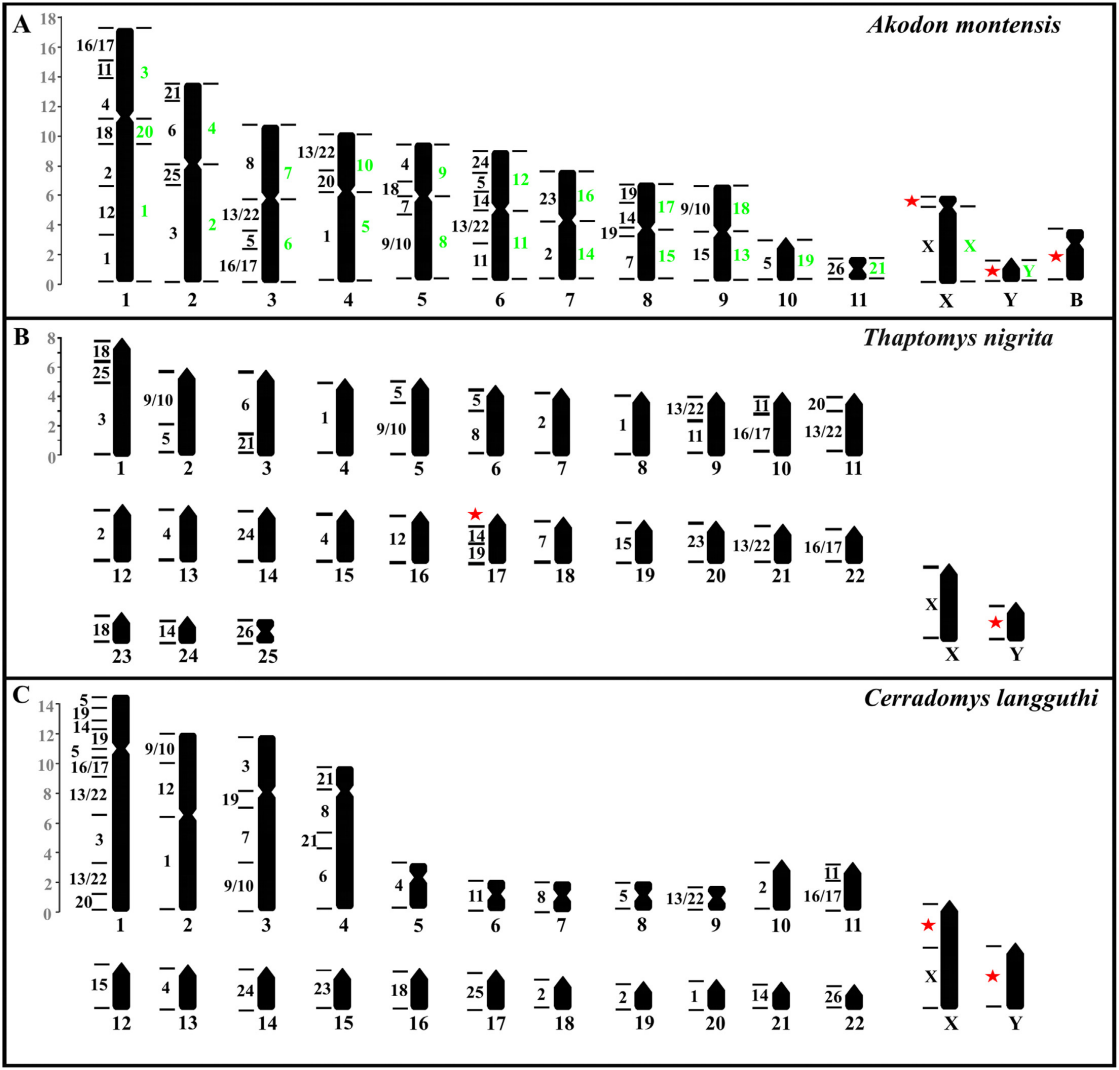
Ideograms of *Akodon montensis* (A), *Thaptomys nigrita* (B), and *Cerradomys langguthi* (C), showing chromosome homologies to *Hylaeamys megacephalus* (left black numbers) and *Akodon paranaensis* (right green numbers). Red stars show regions without complementarity with the probes used. Scale shows the percent relative size of chromosome pairs. (Adapted from Ventura et al. [12], and Nagamachi et al. [17]).

Both Akodontini species show the association of several probes of *H*. *megacephalus* in the same linkage group. Among them, probes 3 and 25, and probes 6 and 21 mapped to two chromosomes of *T*. *nigrita* respectively, but in only one chromosome of *A*. *montensis*, providing evidence of tandem fusion of chromosomes in *A*. *montensis*. Probes 5 and 8; 11 and 13/22; 11 and 16, 17; 20 and 13/22; 14 and 19 were also associated in both species. But as probes 5, 11, 13/22, 14 and 16/17 produce more than one signal in the karyotypes of *T*. *nigrita* and *A*. *montensis*, the synteny relationship of these chromosome fragments may need further confirmation. Table 1 and Fig 3 summarize the complementary relationships revealed by FISH experiments. The X chromosome is the only conserved chromosome both in the taxa studied here and in *Mus musculus*.

## Discussion

**Fig 4:**
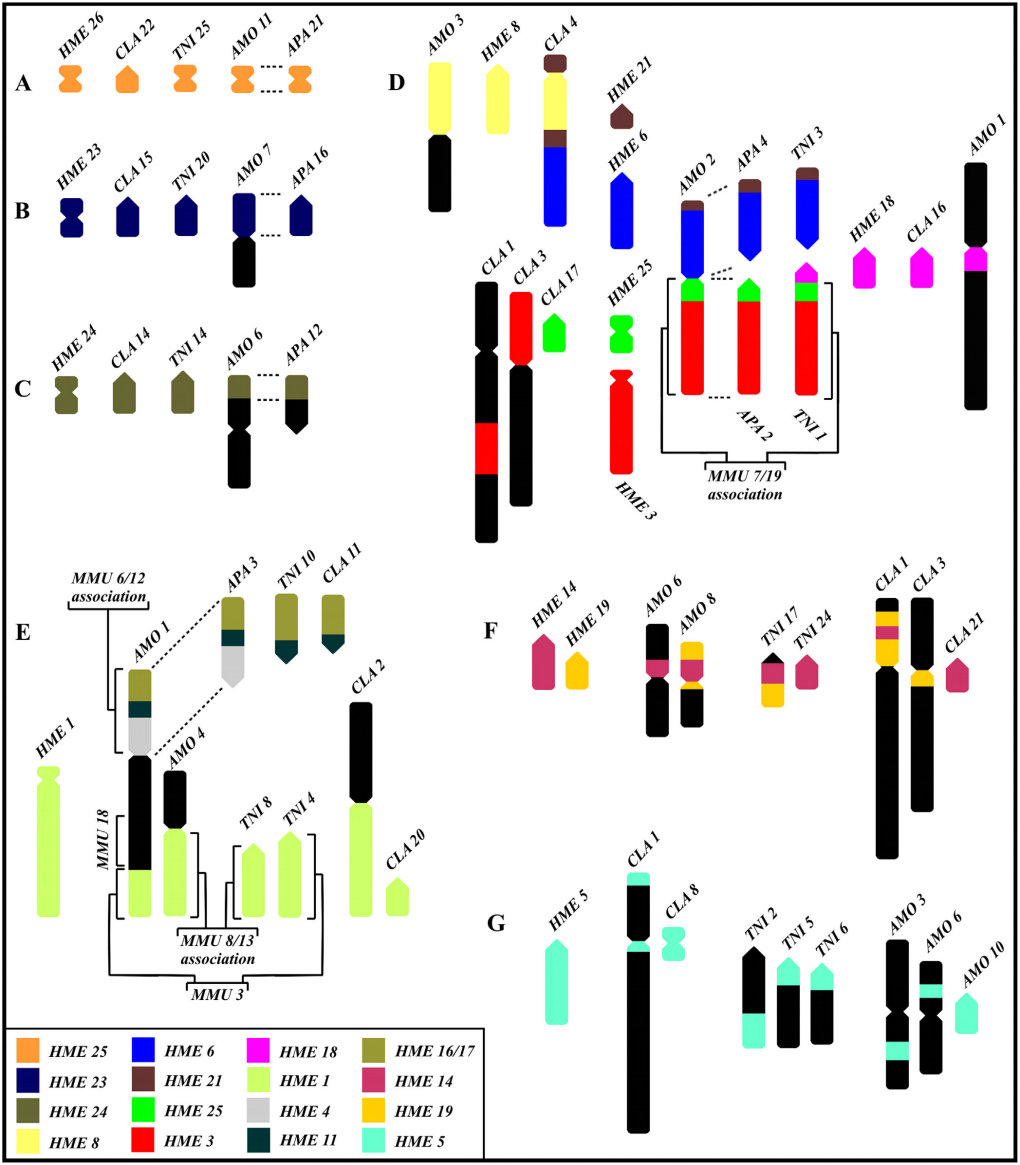
Syntenic relationships among several chromosome pairs of *Hylaeamys megacephalus* (HME), *Akodon montensis* (AMO), *A*. *paranaensis* (APA), *Thaptomys nigrita* (TNI), and *Cerradomys langguthi* (CLA) reveled by different chromosome-paint probes (Adapted from Hass et al [15, 16], Ventura et al. [12], and Nagamachi et al. [17]). Dotted lines: indirectly inferred homology from Ventura et al. [12] data. See Discussion for details.

Bianchi et al. [26] have made the initial cytogenetic hypotheses on chromosome evolution within the Sigmodontinae. Despite methodological constraints, their phylogenetic approach using cytogenetic data group several sigmodontine species by the shared morphology of chromosome 1. They also placed the smallest metacentric pair as a shared characteristic of all karyotyped species. Recently, chromosome painting analyses of four *Akodon* species made by Ventura et al. [13] show the complete homeology of the smallest metacentric pair in *A*. *cursor*, *A*. *montensis*, *A*. *paranaensis*, and *Akodon* sp (2n = 10). The results presented here for *A*. *montensis* and *T*. *nigrita*, and the available data in *Hylaeamys megacephalus* and *Cerradomys langguthi* [18] indicate that this metacentric chromosome pair seems to be the only autosome entirely conserved as an independent linkage group among the Akodontini and Oryzomyini species that have been studied (Fig 4A). However, this chromosome is not completely conserved inasmuch as in *C*. *langguthi* it is telocentric, and this may be a derived condition, due to pericentric inversion or centromeric repositioning. Intriguingly, the same methodological approach, but with *Mus musculus* chromosome probes, fails to identify the homologies of this smallest pair in the sigmodontine species *A*. *cursor*, *A*. *montensis*, *A*. *paranaensis*, *Oligoryzomys flavescens*, *T*. *nigrita*, and *Necromys lasiurus* [16, 17]. These results may be due to technical problems related to its small size, and/or sequence divergence.

Some chromosomes pairs maintain their independent identity despite changes in morphology. Nagamachi et al. [18] describe the relationship among the bi-armed chromosome pairs 23, 24, and 25 of *H*. *megacephalus* (HME23, HME24 and HME25) and their telocentric homologues in C. *langguthi* pairs 15, 14 and 17, respectively (Fig 4B–4D). The results with *H*. *megacephalus* chromosome probes in *A*. *montensis* and *T*. *nigrita*, together with available data from four other *Akodon* species [13], suggest that the bi-armed HME23 and 24 are derived chromosomes, being homologous to the telocentric chromosomes in the two other species. Additionally, the metacentric HME25 is homeologous to the telocentric pair 17 of *C*. *langguthi*.

HME25 is a single pair in Oryzomyini species (*H*. *megacephalus* and *C*. *langguthi*), but is associated with HME3 in *A*. *montensis* (pair 2q) and *T*. *nigrita* (pair 1) (Fig 4D). The association HME25/3 is coincident with the association of *Mus musculus* chromosome pairs 7/19 reported for *A*. *montensis* and *T*. *nigrita* [16, 17], and this has been inferred as an ancestral muroid syntenic association, since it was found in other Cricetidae subfamilies, like Cricetinae [27, 8]. This association already existed before the split of Sigmodontinae and its absence is a derived condition. In *C*. *langguthi* this conserved region is tripartite, probably due to an additional fission of HME3 [18] (Fig 4D). A similar situation is found between HME6 and 21, which are associated in *A*. *montensis* (pair 2p), *T*. *nigrita* (pair 3), and *C*. *langguthi* (pair 4). In the latter there is an intercalary insertion of HME8 [18], splitting the HME21 signal (Fig 4D). In *A*. *montensis* and *T*. *nigrita*, these regions were found to be homeologous to *M*. *musculus* chromosome 2 [16, 17].

The association HME25/3/18 in *T*. *nigrita* could represent a putative autapomorphy (Fig 4D). Their close phylogenetic relationship with *Akodon serrensis* [28, 29], in which chromosome pair 1 is apparently derived from a chromosome fusion [6], needs to be more closely evaluated.

The *Mus musculus* association 8/13 was suggested as a putative synapomorphy for South American Cricetidae [16, 17]. Its presence in *A*. *montensis* [16] and *T*. *nigrita* [17] corresponds to one of the two signals observed with HME1 (Fig 4E). The second signal of this probe corresponds to the *M*. *musculus* chromosome probe of pair 3 in *A*. *montensis* (pair 1) and *T*. *nigrita* (pair 8), which strongly suggests that the *H*. *megacephalus* chromosome pair 1 is the result of a fusion event between the homeological regions of the highly conserved *M*. *musculus* chromosome pair 8/13 association and the *M*. *musculus* chromosome pair 3. This possibility had already emerged from a comparative G-banding pattern analysis between *H*. *megacephalus*, *H*. *yunganus* and *Euryoryzomys nitidus* (pairs 7, 4, and 8, respectively) [30].

The associations of chromosome pair 3/18 and 6/12 of *M*. *musculus* have been postulated as synapomorphies in Akodontini [16, 17]. The first association co-localized mainly with chromosome pair 1 of those *Akodon* species with karyotypes composed mainly of telocentric chromosomes (*A*. *paranaensis* and *A*. *serrensis*), the distal half of the long arm of pair 1 of *A*. *montensis* (Figs 3A and 4E), and on chromosome pair 5 of *Necromys lasiurus*, but is not found in *T*. *nigrita* [13, 17]. The second association seems to be restricted to chromosome 3 of *A*. *paranaensis* and *A*. *serrensis* [16], to their homeological regions of other *Akodon cursor* species group [13], and to pair 10 of *N*. *lasiurus* [17]. This association corresponds to the association of HME4/11/16/17 on the short arm of chromosome 1 of *A*. *montensis* (Fig 4E). However, in *T*. *nigrita* this is not fully preserved since a portion is co-localized on pair 10 associated with *H*. *megacephalus* chromosome pairs 11/16/17 (Fig 4E). Interestingly, this association (11/16/17) was also found in *C*. *langguthi* on chromosome pair 11 [18] (Figs 3C and 4E).

*Hylaeamys megacephalus* pair 14 probe paints two different chromosomes, one of which appears associated with HME19 in *A*. *montensis*, *T*. *nigrita*, and *C*. *langguthi* (Fig 4F) [18]. The fission seems to be a derived condition on *H*. *megacephalus* karyotype. In *A*. *montensis* and *T*. *nigrita*, the chromosome regions where FISH signals were observed for both probes are co-localized with *M*. *musculus* chromosome pair 11 [16, 17]. The additional signal observed for HME19 in *A*. *montensis* and *Cerradomys langguthi* could be explained by an inversion event, but it is necessary to corroborate whether this is one or two independent events.

The *H*. *megacephalus* chromosome probe 5 is the most fragmented chromosome pair in *A*. *montensis*, *T*. *nigrita*, and *C*. *langguthi* (Fig 4G). One interpretation of these results could be related to chromosomal fragmentation in the evolutionary pathway of *Cerradomys*, *Thaptomys* and *Akodon* species. However, a more parsimonious alternative is that it could be the outcome of the fusions of three independent syntenic units that derived from chromosome pair 5 of *H*. *megacephalus*.

The X chromosomes were the only syntenic units that are conserved as a unique and independent linkage group in all sigmodontine species studied and in *Mus musculus*. The high conservation of this sex chromosome in the rodent lineage, as in others mammals, was detected by several investigations [31]. However, some variations of its morphology were described. In *A*. *montensis*, the heteromorphism observed on the X chromosomes appears to be the result of the addition of heterochromatin in its short arm. This is supported by the absence of hybridization with the *H*. *megacephalus* probes and by the observation of a C positive band in its short arm (Fig 1A black square). The results presented by Nagamachi et al. [18] using the same probe of chromosome X of *H*. *megacephalus* has been useful also in distinguishing the additional constitutive heterochromatin on the X chromosome of *Cerradomys langguthi*.

A set of associations (*H*. *megacephalus* chromosome pairs 6/21, 11/16,17, and 14/19) has shown interesting synapomorphies among the Akodontini and Oryzomyini tribes. Extend these observations to other members of both tribes is necessary to confirm the phylogenetic assumptions presented here and to improve our knowledge of the chromosome evolution of these two important groups of Neotropical rodent fauna.

## Supporting Information

S1 Fig Giemsa and DAPI stained chromosomes pairs of *Akodon montensis* with respective signals of each specific-chromosome probes of *Hylaemys megacephalus*. (JPG)

S2 Fig Giemsa and DAPI stained chromosome pairs of *Thaptomys nigrita* with respective signals of each specific-chromosome probes of *Hylaemys megacephalus*. (JPG)
