# Supplementary figures and images for: Clues on Syntenic Relationship among Some Species of Oryzomyini and Akodontini Tribes (Rodentia: Sigmodontinae)
Source: PLoS One. 2015 Dec 7;10(12):e0143482. doi: 10.1371/journal.pone.0143482 (PMC4671618; doi:10.1371/journal.pone.0143482)

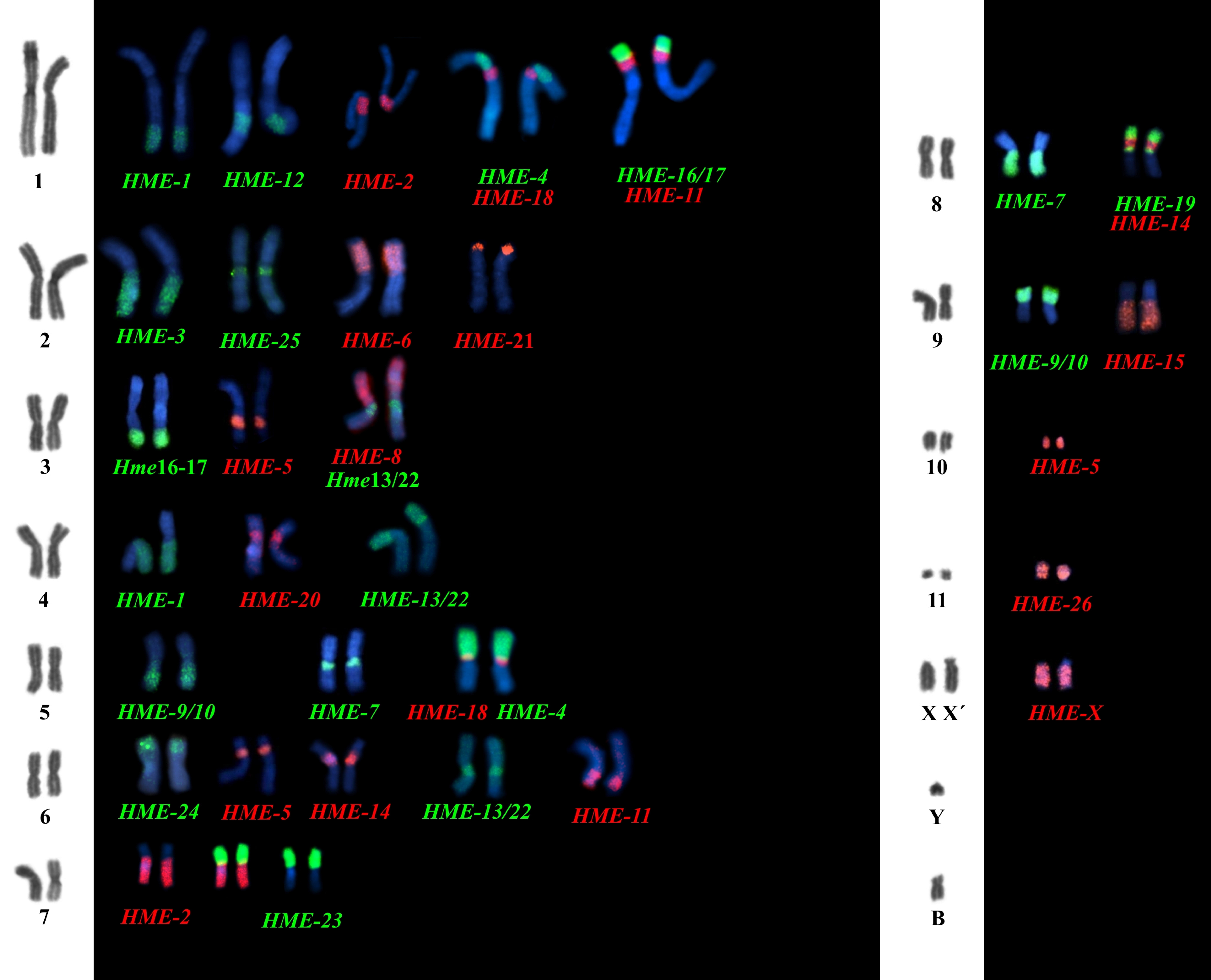

Supplement: S1 Fig — (JPG) [file pone.0143482.s001.jpg]

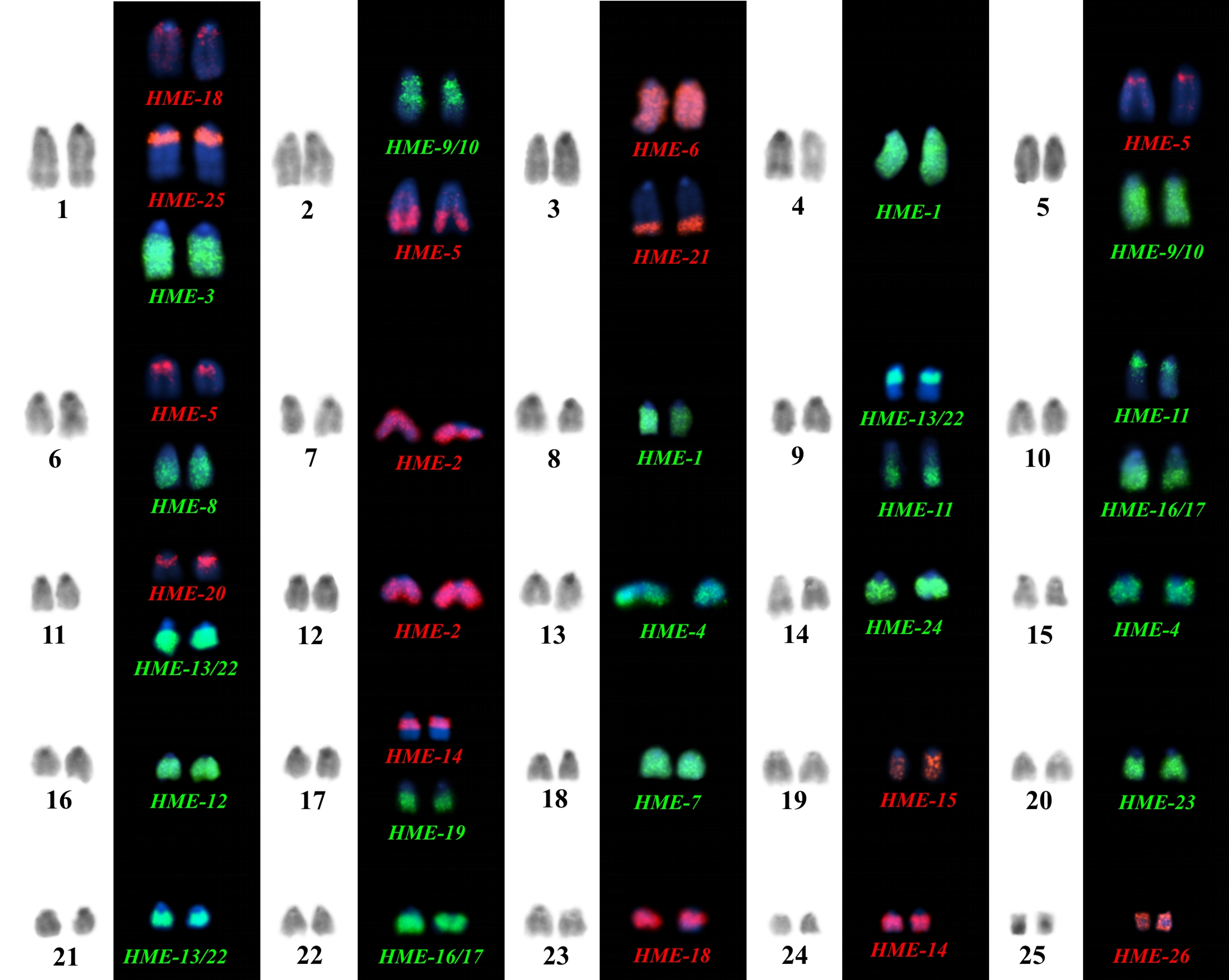

Supplement: S2 Fig — (JPG) [file pone.0143482.s002.jpg]
